# Peripheral Arterial Disease among Adult Diabetic Patients Attending a Large Outpatient Diabetic Clinic at a National Referral Hospital in Uganda: A Descriptive Cross Sectional Study

**DOI:** 10.1371/journal.pone.0105211

**Published:** 2014-08-18

**Authors:** Raymond Mbayo Mwebaze, Davis Kibirige

**Affiliations:** 1 Department of Medicine and Diabetes/endocrine clinic, St. Raphael of St. Francis hospital Nsambya, Kampala, Uganda; 2 Mother Kevin Postgraduate Medical School, Uganda Martyrs University, Nkozi, Uganda; 3 Department of Medicine, Uganda Martyrs Hospital Lubaga, Kampala, Uganda; Medical University Innsbruck, Austria

## Abstract

**Background:**

Peripheral arterial disease (PAD) is one of the recognised diabetic macro vascular complications. It is a marker of generalised systemic atherosclerosis and is closely associated with symptomatic coronary and cerebrovascular disease, hence significant morbidity and mortality. Among African adult diabetic populations, screening and diagnosis of PAD is frequently suboptimal. The aim of this study was to determine the prevalence and associated clinical factors of PAD in adult ambulatory diabetic patients attending the outpatient diabetic clinic of Mulago national referral and teaching hospital, Kampala Uganda.

**Methods:**

In this descriptive cross sectional study, 146 ambulatory adult diabetic patients were studied. Information about their socio-demographic and clinical characteristics, fasting lipid profile status, blood pressure, glycated haemoglobin (HbA1c) levels and presence of albuminuria was collected using a pre tested questionnaire. Measurement of ankle brachial index (ABI) to assess for PAD, defined as a ratio less than 0.9 was performed using a portable 5–10 MHz Doppler device. Clinical factors associated with PAD were determined by comparing specific selected characteristics in patients with PAD and those without.

**Results:**

The mean age/standard deviation of the study participants was 53.9/12.4 years with a male predominance (75, 51.4%). PAD was prevalent in 57 (39%) study participants. Of these, 34 (59.6%) had symptomatic PAD. The noted clinical factors associated with PAD in this study population were presence of symptoms of intermittent claudication and microalbuminuria.

**Conclusions:**

This study documents a high prevalence of PAD among adult ambulatory Ugandan diabetic patients. Aggressive screening for PAD using ABI measurement in adult diabetic patients should be emphasised in Uganda especially in the presence of symptoms of intermittent claudication and microalbuminuria.

## Introduction

Cardiovascular diseases (CVD) are a major cause of morbidity and mortality in adult patients with diabetes mellitus (DM). Macro vascular complications like stroke, myocardial infarction and PAD tend to occur earlier in diabetics compared to non diabetics [1].

PAD is a manifestation of widespread systemic atherosclerosis characterized by atherosclerotic occlusive disease of the lower extremities and is one of the macro vascular complications highly prevalent in adult diabetic patients. The majority of diabetic patients with PAD have concomitant coronary artery disease [2], [3] and a significant burden of morbidity and mortality in these patients is intimately associated with myocardial infarction, ischemic stroke or sudden cardiovascular death [4].

Epidemiologic data has shown a strong association between PAD and DM. Other notable traditional risk factors of PAD include smoking, increasing age, hypertension and hyperlipidemia and ethnicity (black race) [5].

In Sub Saharan Africa (SSA), the prevalence of PAD varies from 1.7–52.5% [6], [7], [8], [9], [10], [11], [12], [13] with higher prevalence noted in studies that use Doppler based means of diagnosis as opposed to clinical evaluation (inspection for foot colour/skin changes and palpation for absent/diminished foot pulses). Despite this varied prevalence, screening of PAD among adult diabetic patients in SSA is infrequent [11], [13].

This implies that a significant proportion of diabetic patients remain undiagnosed and are at risk of foot complications like foot ulceration and gangrene and CVD. Early screening, detection and optimal management of asymptomatic PAD among the African adult diabetic population would significantly lower subsequent morbidity and mortality and systemic atherosclerotic diseases.

Screening for PAD by measuring the ABI, which is the ratio of the tibial systolic artery pressure to brachial systolic artery pressure is preferred to clinical inspection of lower limbs and palpation of the feet pulses. ABI measurement is a non-invasive procedure performed using a Doppler ultra sound device and has a 95% sensitivity and 100% specificity for diagnosing PAD compared to the gold standard angiography. An ABI of less than 0.9 is diagnostic of PAD. The normal range is between 0.9 and 1.1 while values ≥1.3 signify non compressible or calcified peripheral arteries [14].

In Uganda, there is dearth of studies examining the burden of PAD and related clinical factors in adult diabetic patients. We therefore conducted this descriptive cross sectional study among 146 eligible adult diabetic patients aged ≥30 years attending the diabetic outpatient clinic of Mulago national referral and teaching hospital in a 3 month period.

## Methods

### Study setting

This study was conducted at the diabetic outpatient clinic of Mulago national referral and teaching hospital, Kampala Uganda. It is a follow up clinic where new and old diabetic patients are reviewed once a week. It is operated by 3 physicians, 4 internal medicine residents and 3 diabetic nurses, all attached to the diabetes/endocrine unit of the hospital. An average of 60 old patients and 10 new patients are seen every clinic day.

### Study population and sampling procedure

The study population composed of 146 adult Ugandan ambulatory diabetic patients aged ≥30 years regularly attending the outpatient diabetic clinic for at least 1 year.

Systematic random sampling was used to recruit the patients into the study. This was done by choosing the 5th patient as they registered during the clinic day. In case the patient declined to participate in the study or was ineligible, the next one was selected. This was done every clinic day until the desired sample size was attained.

The inclusion criteria into the study were: an ambulatory diabetic patient aged ≥30 years who had been regularly attending the diabetic outpatient clinic at this institution for at least 1 year and diabetic patients who had offered informed consent to participate in the study.

Adult diabetic patients with serious co-morbid medical conditions like terminal HIV infection, advanced malignancies were excluded from the study. A convenience sample size of 146 subjects was used in this study.

### Data collection

Pre tested and pre coded questionnaires were used to collect the study information which included: socio demographic characteristics (age, gender, educational level, occupation, religion, address, smoking status, alcohol intake) and clinical history (age at initial diagnosis of DM, duration of DM, current medical therapy, history of hypertension (HT) and history of symptoms of intermittent claudication).

Physical examination findings like the blood pressure (BP), weight and height for calculation of the body mass index (BMI), presence and quality of the lower limb pulses on palpation, presence of ulcers/gangrene on inspection and the calculated ABI were also documented. All patients underwent laboratory measurement of the HbA1c, fasting lipid profile and assessing for albuminuria on a spot mid stream urine sample using standardised methods.

The questionnaire was administered to the study participants after offering informed consent by the trained research assistants. The above stated socio demographic and clinical characteristics were entered into the questionnaire. To document the presence or absence of the symptoms of intermittent claudication i.e. leg-muscle discomfort on exertion that is relieved with rest [15], the Edinburgh intermittent claudication questionnaire was used. In one study, this questionnaire was found to be 91.3% (95% CI 88.1–94.5%) sensitive and 99.3% (95% CI 98.9–100%) specific in comparison to the diagnosis of intermittent claudication made by a physician in one study. Its repeatability after 6 months was excellent (kappa = 0.76, *p*<0.001) [16].

All patients had standard anthropometric measurements of height in metres (m) and weight in kilograms (kg) for calculation of BMI using the formula: BMI = weight in kg/height in m^2^. Blood pressure was measured in both arms using a mercury sphygmomanometer after a 15 minute rest. The average value was recorded as the patient's BP.

A detailed physical examination of the peripheral lower limb was also performed (palpation for quality of foot pulses and clinical inspection for any skin colour, hair and nail changes, temperature variations and for presence of ulcerations and gangrene).

A resting ABI of each lower limb was then determined in supine position using a portable Doppler machine with a VP5HS-5MHz probe for deep lying vessels and oedematous limbs and EZ8 " wide beam" probe for peripheral vessels. Blood pressure cuffs were placed bilaterally on the upper arm (brachial pressure) and at the ankle just above the medial malleoli.

An ultrasound transducer was used to locate the arterial Doppler signals distal to the blood pressure cuffs. The Doppler signal from the brachial artery was used to obtain the arm pressure while that from the dorsalis pedis and posterior tibial arteries was used to obtain the ankle pressure. The higher systolic pressure of the anterior dorsalis pedis or posterior tibial measurement for each foot was divided by the highest brachial systolic pressure to obtain the ABI for each limb.

A mid stream urine sample was obtained from the study participants to assess for presence of albuminuria (microalbuminuria and macroalbuminuria). Six mls of venous blood were drawn for assessment of the HbA1c and fasting lipid profile. Prior to the above tests, patients had to be fever free, fasted overnight for at least 8–12 hours, had not smoked or engaged in any physical activity. All the laboratory analysis was done at the clinical chemistry laboratory of Mulago national referral and teaching hospital, Uganda.

### Study definitions

PAD was defined as an ABI≤0.9. Mild, moderate and severe obstruction were defined as ABIs of 0.7–0.9, 0.4–0.69 and <0.4 respectively. A value of 0.91–1.3 was defined as a normal ABI while an ABI >1.3 signified poorly compressible arteries or arterial calcification. Normal BMI, overweight and obesity were defined as BMI of 18–24.9, 25–29.9 and ≥30 kg/m2 respectively. Hypertension was defined as a blood pressure of ≥140/90 mmHg or being on anti hypertensive treatment.

### Statistical analysis

Data was coded and double entered in EpiData, cleaned and exported to STATA10 for analysis. The prevalence of PAD was calculated as the percentage of study participants with ABI ≤0.9 and the total study sample size being the denominator. The results for the descriptive statistics were presented using frequency tables, pie chart and means±standard deviations.

The significant clinical factors associated with PAD were obtained by comparing specific patient characteristics between those with PAD and those without. A p value of ≤0.05 was considered as statistically significant.

### Ethical consideration

Ethical approval to conduct this study was obtained from the department of medicine, College of Health Sciences and the research and ethics committee School of Medicine, Makerere University Kampala Uganda. All the study participants provided written informed consent to participate in this study.

## Results

### Socio demographic and clinical characteristics of study participants

Of the 146 patients recruited in the study, majority were males (75, 51.4%) giving a male: female ratio of 1.1∶1. The mean age/standard deviation of the study participants was 53.9/12.4 years with the youngest and oldest being 30 years and 81 years respectively. Eighty seven (59.6%) participants were ≥50 years.

Only 24 (16.4%) participants drunk alcohol, however in the moderate recommended amounts. The CAGE assessment for alcohol abuse among all these patients was negative. Majority of the study participants were non smokers (96.6%). Only 1 participant was currently smoking and 2 were former smokers.

The mean age of diagnosis of DM in the study participants was 48.1±7.2 years with the minimum and maximum age at diagnosis of 20 years and 79 years respectively. The greatest proportion of participants had short duration of DM of 0–4 years (74, 50.7%). The mean duration of DM was 5.75±3.21 years.

Apart from 2 (1.4%) participants who were on conservative management using diet and exercise, the rest received pharmacological therapy for glucose lowering. Majority were receiving oral hypoglycaemic therapy [sulphonylureas and metformin combination] (79, 54.1%) while 55 (36.7%) participants were on insulin either as monotherapy or in combination with metformin.

Pre-existing hypertension was documented in 69 (47.3%) participants, of which only 1 of these had optimal BP of ≤140/80 mmHg. Among the participants without a previous or known diagnosis of hypertension, hypertension as defined as BP ≥140/90 mmHg was documented in 49 (63.6%) patients. Statin and anti platelet therapy was noted in only 12% and 22% of study participants respectively.

Table 1: summarises the socio demographic and clinical characteristics of the study participants.

**Table 1:** Socio demographic and clinical characteristics of the study participants.

Characteristic	Frequency	Percentage
**Age, years**		
Mean±SD: 53.9±12.4 years		
≥50	87	59.6
**Gender**		
Males	75	51.4
**Occupation**		
Employed	122	83.6
**Education status**		
No formal education	26	17.8
Primary education	61	41.8
Secondary education	46	31.5
Tertiary education	13	8.9
**Smoking status**		
Former smokers	2	1.4
Current smokers	1	0.7
Alcohol consumption	24	16.4
**Age of onset of diabetes**		
≥40 years	102	69.9
**Duration of DM**		
≤4 years	74	50.7
Pre-existing hypertension	69	47.3

### Clinical examination findings of the study participants

Normal BMI, overweight and obesity were documented in 43.2%, 26.7% and 27.4% respectively. The quality of the pulses of the lower limbs on clinical palpation were graded as 0, 1 and 2 when absent, diminished and normal. Diminished pulses were noted in the popliteal, dorsalis pedis and posterior tibial arteries in proportions of 10%, 28.8% and 29.5% respectively. None of the patients had absent foot pulses. Therefore, using the criteria of palpation of all those lower limb pulses, the prevalence of PAD would be 22.9%.

On clinical inspection and palpation of the lower limbs, presence of thickened nails, low temperature (cold feet) and atrophy of the subcutaneous fat were the most prevalent clinical findings in 18 (12.3%) and 13 (8.9%) study participants respectively. Shiny skin and hair loss was noted in 5.5% and 2.1% of the study participants respectively. Table 2 summarises the clinical examination findings.

**Table 2:** Summarises the clinical examination findings.

Characteristic		Frequency	Percentage (%)
**Body mass index in kg/m^2^**			
<18		4	2.7
18–24.9		63	43.2
25–29.9		39	26.7
>30		40	27.4
**Pulse**	**Pulse quality**	**Frequency**	**Percentage**
Femoral	Normal	146	100
Popliteal	Diminished	15	10.3
	Normal	131	89.7
Dorsalis pedis	Diminished	42	28.8
	Normal	104	71.2
Posterior tibial	Diminished	43	29.5
	Normal	103	70.5
**Summary of lower limb examination findings**			
**Symptom or sign**		**Frequency**	**Percentage (%)**
Intermittent claudication		65	44.5
Diminished foot pulses		33	22.9
Thickened nails		18	12.3
Cold feet		13	8.9
Atrophy of subcutaneous fat		13	8.9
Shiny skin		8	5.5
Loss of hair		3	2.1
Rest pain		2	1.4
Nocturnal pain		1	0.7
Ulcers and/gangrene		0	0

### Laboratory findings of the study participants

Table 3 summarises the various laboratory findings of the study participants. Optimal glycaemic control defined as HbA1c of ≤7% [17] was observed in 28 (19.2%) participants. The mean HbA1c was 9.49±2.52%. Optimal low density Lipoprotein cholesterol (LDLC) levels of ≤100 mg/dl, triglyceride levels ≤150 mg/dl, total cholesterol ≤200 mg/dl and high density lipoprotein cholesterol (HDLC) ≥60 mg/dl [17] were documented in 48.6%, 95.8%, 79.5% and 11.6% respectively. Microalbuminuria and macroalbuminuria was noted in 39.7% and 8.9% respectively.

**Table 3:** Summarises the various laboratory findings of the study participants.

Laboratory finding	Frequency	Percentage (%)
**HbA1c in %**		
≤7	28	19.2
>7	118	80.8
**Total cholesterol in mg/dl**		
≤200	116	79.5
>200	30	20.5
**LDLC in mg/dl**		
≤100	71	48.6
>100	75	51.4
**HDLC in mg/dl**		
≥60	17	11.6
<60	129	88.4
**Triglycerides in mg/dl**		
≤150	140	95.8
>150	6	4.2
**Albuminuria**		
No albuminuria	75	51.4
Micro albuminuria	58	39.7
Macro albuminuria	13	8.9

### PAD and intermittent claudication-the prevalence

Using the ABI measurement, a low ABI of ≤0.9 to define the presence of PAD was noted in 57 participants, giving a prevalence of 39% with an observed male preponderance (33, 57.9%). With the exception of 1 participant who had moderate obstruction of the left limb, the rest of the participants had mild obstruction (99.3%). No study participant had severe PAD while 5 (3.4%) participants had poorly or non compressible vessels.

Using the Edinburgh intermittent claudication questionnaire in the 146 study participants, 65 (44.5%) had definite claudication. Of these, 34 (52.3%) participants had both symptoms of intermittent claudication and PAD (a low ABI ≤0.9) and 31(47.7%) had symptoms of intermittent claudication without PAD (ABI ≥0.9).

### Clinical factors associated with PAD

Table 4 shows the correlation of PAD with specific selected socio demographic, clinical and laboratory characteristics. Presence of intermittent claudication and microalbuminuria were associated with PAD in this study population. A low HDLC concentration showed a trend towards significance.

**Table 4:** Association of PAD with specific selected socio demographic, clinical and laboratory characteristics.

Characteristics	PAD (n = 57, 39%)	No PAD (n = 89, 61%)	P value
**Socio demographic**			
Age ≥50 years	34 (39.1)	53 (60.9)	0.991
Male gender	33 (44)	42 (56)	0.207
**Clinical characteristics**			
Age of onset of DM (≥40 years)	39 (38.2)	63 (61.8)	0.761
Duration of DM (≥10 years)	39 (38.2)	63 (61.8)	0.761
Obesity as per BMI	17 (42.5)	23 (57.5)	0.12
Hypertension	44 (37.6)	73 (62.4)	0.19
***Intermittent claudication***	***34 (52.3)***	***31 (47.7)***	***0.03***
**Laboratory findings**			
HbA1c ≥8%	39 (39)	61 (61)	0.988
LDL cholesterol ≥130 mg/dl	10 (31.3)	22 (68.8)	0.539
HDL cholesterol ≤40 mg/dl	30 (44.8)	37 (55.2)	0.056
Triglycerides ≥200 mg/dl	2 (33.3)	4 (66.7)	0.770
***Micro albuminuria***	***33 (56.9)***	***25 (43.1)***	***0.01***

## Discussion

To our knowledge, this is the study to examine the burden of PAD and associated clinical factors in adult Ugandan diabetic patients using ABI measurement. We report a prevalence of PAD of 39% in this study population with preponderance in male patients (57.8%). Majority of the study participants had symptomatic PAD (59.6%) and mild obstruction (99.3%) according to the ABI measurement. Asymptomatic PAD was noted in 41.4% of the study participants. The presence of symptoms of intermittent claudication and microalbuminuria were significantly associated with PAD.

This documented prevalence is higher than what is reported from other regions outside Africa. Data from the Framingham Heart study revealed a prevalence of symptomatic PAD of 20% in adult diabetic patients [18]. In the Bypass Angioplasty Revascularization Investigation 2 Diabetes (BARI 2D) Trial another large study to examine the magnitude of ABI abnormalities in 2,240 diabetic patients with coronary artery disease, low ABI was found in 19% of the patients [3]. PAD in this study was independently associated with smoking, female gender, black race, hypertension, increasing age, C-reactive protein, diabetes duration, and lower BMI.

In another multi racial cross sectional study performed in Malaysia, Asia to determine the prevalence of ABI in 200 diabetic patients at a primary care setting, the overall prevalence of PAD was 16% in this diabetic population. The prevalence of PAD was 5.8% in Malays, 19.4% in Chinese and 19.8% in Indians. However, no significant relationships were found between age, gender, smoking status, duration of diabetes mellitus, hypertension, dyslipidemia, and PAD [19].

In SSA, different prevalence of PAD ranging from 1.7–52.5% have been reported in a literature review and most studies [6], [7], [8], [9], [10]. This can be explained by the varied study diagnostic approaches of PAD with higher prevalence noted in studies using ABI measurement [7], [8], [9], [10] compared to clinical palpation of lower limb pulses [11], [12], [13].

With the exception of a similar study from Nigeria [7], the prevalence of PAD in this study (39%) is higher than what has been documented from other African studies who used a similar diagnostic approach [8], [9], [10]. Studies from Tanzania by Gulam-Abbas Z et al [9] and South Africa by Paul A et al [8] and Rheeder P [10] et al reported prevalence of 20.7%, 29.3% and 4.7% respectively. A prevalence of 52.5% was noted in the Nigerian study [7]. This higher prevalence in the Nigerian study could probably be explained by the recruitment of only older diabetic patients between the ages of 50–89 years.

The plausible explanations for the observed high prevalence of PAD in our study include: high prevalence of pre existing hypertension (47.3%), obesity (27.4%) and overweight (26.7%), low frequency of use of lipid lowering drugs (statins) [12%] and anti-platelet drugs (22%) and suboptimal glycaemic, blood pressure and lipid control among the study population. Of the 69 diabetic patients with HT co-morbidity, only 1(1.5%) had optimal BP control as per the ADA guidelines [17]. Optimal glycaemic control was noted in only 19.2% of the study participants. Low HDLC levels an integral component of metabolic syndrome and high LDLC levels were highly prevalent in this study population with 88.4% and 51.4% of participants having suboptimal concentrations according to the same ADA guidelines.

Despite the high prevalence of PAD in this study population, mild arterial obstruction was noted in a large proportion of patients (145, 99.3%). Clinical inspection of the lower limbs did not reveal any presence of vascular ulcers and gangrene. This high prevalence of mild PAD could probably be explained by the very low rate of smoking (0.7%), moderate alcohol ingestion (16.4%) and high prevalence of short duration of DM i.e. ≤4 years (50.7%) among the study participants.

Measurement of ABI is one of the most recommended approaches of diagnosing PAD due to its non invasiveness and high sensitivity and specificity [14]. Clinical diagnosis basing on presence of symptoms of intermittent claudication and or palpation for diminished or absent lower limb pulses is very unreliable and misses most cases of PAD.

In this study, it worth noting that symptoms of intermittent claudication were present in 47.7% of study participants with a normal ABI. The use of clinical palpation of all the lower limb pulses for diminished or absent pulses would diagnose fewer cases of PAD in this study population (22.9%). This finding is congruent to what was also observed in the Nigerian study where clinical palpitation for absent lower limb pulses diagnosed PAD in only 11.4% compared to 52.5% with ABI measurement [7].

Another retrospective study examining the quality of diabetes care among adult diabetics at a large outpatient diabetic clinic in a private hospital in Uganda also found a very low prevalence of PAD of 2% basing on clinical examination of quality of foot pulses [11].

The presence of symptoms of intermittent claudication and microalbuminuria were significantly associated with PAD. Intermittent claudication defined as leg-muscle discomfort on exertion that is relieved with rest is a classical feature of PAD [15]. However, due to presence of diabetic neuropathy that causes reduced sensation, a significant proportion of patients with PAD are usually asymptomatic. In this study, 41.4% of the patients with PAD were asymptomatic.

Presence of microalbuminuria was also associated with PAD in this study population. Compelling evidence recognises microalbuminuria as one of the principal predictive factors of cardiovascular complications, all cause and cardiovascular mortality independent of the traditional risk factors like dyslipidemia, hypertension in diabetics, hypertensives and general population. The pathophysiologic mechanisms that link albumin excretion and CVD are not fully defined.

The plausible theories are that microalbuminuria may be a marker of CVD risk because it reflects subclinical vascular damage in the kidneys and other vascular beds. It may also signify widespread systemic endothelial dysfunction and an increased pro inflammatory state that predisposes to atherosclerosis and potential cardiovascular events [20], [21], [22].

No African study has observed an association between microalbuminuria and PAD. Increasing age and being widowed were associated with PAD in the Nigerian study [7]. The risk of CVD increases with age while limited financial support to access appropriate medical care in the widowed patient category was thought to explain that association. Smoking and male gender were associated with PAD in one of the South African studies [8].

## Implications of the Study

This study evidently demonstrates a high prevalence of PAD of 39% in our adult ambulatory diabetic patients attending the outpatient diabetic clinic at Mulago national referral and teaching hospital, Kampala Uganda. It also reveals that a section of patients with PAD can be asymptomatic. Diagnosing PAD basing on presence of diminished or absent foot pulses on clinical palpation of foot pulses misses a reasonable proportion of patients, hence ABI measurement as a means of diagnosis should be universally adopted in all the adult diabetic clinics as a more superior screening approach in Uganda.

There is need to provide sufficient portable Doppler machines in our diabetic centres and also extensively train health workers involved in diabetes care to screen for PAD in adult diabetics especially those with symptoms of intermittent claudication and microalbuminuria. Regular national or regional awareness and training seminars for health workers and patients should be introduced with an aspiration of improving diabetes care i.e. ensuring optimal glycaemic, blood pressure and lipid control, screening for macro and micro vascular complications and appropriate primary and secondary prophylaxis against CVD.

## Study Limitations

Due to the small size of the study and being hospital based, these findings cannot be generalised to the general population. This study was performed among diabetic ambulatory patients and this may have lead to an underestimation of the problem since many patients with the more serious diabetic complications are either admitted or managed by the vascular surgeons. We were unable to perform pulse oximetric toe pressures for patients with poorly compressed vessels.

## Conclusions

The overall prevalence of PAD in this study population was 39% with presence of symptoms of intermittent claudication and microalbuminuria being significantly associated with PAD. We recommend routine screening of adult diabetic patients for PAD using ABI measurement in Uganda and SSA universally.
